# Retinoid homeostasis in major depressive disorder

**DOI:** 10.1038/s41398-023-02362-0

**Published:** 2023-02-23

**Authors:** Lisa Rebecca Otto, Vera Clemens, Berk Üsekes, Nicoleta Carmen Cosma, Francesca Regen, Julian Hellmann-Regen

**Affiliations:** Charité—Universitätsmedizin Berlin, corporate member of Freie Universität Berlin and Humboldt-Universität zu Berlin, Department of Psychiatry and Psychotherapy, Section Clinical Neurobiology, Campus Benjamin Franklin, Berlin, Germany

**Keywords:** Biomarkers, Depression

## Abstract

The small, hormone-like molecule retinoic acid (RA) is a vital regulator in several neurobiological processes that are affected in depression. Next to its involvement in dopaminergic signal transduction, neuroinflammation, and neuroendocrine regulation, recent studies highlight the role of RA in homeostatic synaptic plasticity and its link to neuropsychiatric disorders. Furthermore, experimental studies and epidemiological evidence point to the dysregulation of retinoid homeostasis in depression. Based on this evidence, the present study investigated the putative link between retinoid homeostasis and depression in a cohort of 109 patients with major depressive disorder (MDD) and healthy controls. Retinoid homeostasis was defined by several parameters. Serum concentrations of the biologically most active Vitamin A metabolite, *all-trans* RA (*at-*RA), and its precursor retinol (ROL) were quantified and the individual in vitro *at-*RA synthesis and degradation activity was assessed in microsomes of peripheral blood-derived mononuclear cells (PBMC). Additionally, the mRNA expression of enzymes relevant to retinoid signaling, transport, and metabolism were assessed. Patients with MDD had significantly higher ROL serum levels and greater *at-*RA synthesis activity than healthy controls providing evidence of altered retinoid homeostasis in MDD. Furthermore, MDD-associated alterations in retinoid homeostasis differed between men and women. This study is the first to investigate peripheral retinoid homeostasis in a well-matched cohort of MDD patients and healthy controls, complementing a wealth of preclinical and epidemiological findings that point to a central role of the retinoid system in depression.

## Introduction

Depression is a complex and debilitating disorder affecting roughly 300 million people worldwide and exacting huge tolls on individuals and societies [1]. Given the multifactorial etiology and heterogeneous phenotypic expressions of the disorder, there is a vast number of concepts on depression pathophysiology. Alterations in major neurobiological processes such as monoamine neurotransmission, neuroendocrine regulation, inflammatory processes, and particularly multiple forms of cellular and synaptic plasticity have been described for depression [2, 3]. Accordingly, several targeted treatments have been developed, mainly targeting the level of monoaminergic and serotonergic neurotransmission. However, despite tremendous progress in elucidating neurobiological correlates, the final, individual, and possibly patient-specific molecular mechanisms and their reciprocal interactions are still incompletely understood [4, 5]. Modulating effects on neural plasticity at levels beyond direct neurotransmitter actions have recently been described for the hormone-like small molecule retinoic acid (RA) [6].

RA—the active metabolite of vitamin A (retinol, ROL)—is a highly potent neural regulator that acts in an auto- and paracrine manner and is involved in a range of brain physiological processes. While RA has previously mostly been considered in the context of embryonic and early postnatal development, its important role in the healthy functioning of the adult brain is now well established [7]. Studies investigating its molecular actions revealed a remarkable involvement of RA in essential neurobiological pathways that are also affected in neuropsychiatric disorders. For example, RA was shown to be a crucial component in the signal transduction pathways of dopaminergic and neuropeptide signaling, neuroinflammation, and hypothalamic neuroendocrine regulation [8–13]. Furthermore, several aspects of neuronal plasticity such as neurite outgrowth, neurogenesis, and Hebbian forms of synaptic plasticity rely on RA signaling [14–17]. Strikingly, RA also functions as a critical regulator during homeostatic synaptic plasticity which is a non-Hebbian form of plasticity and serves as a mechanism of metaplasticity to maintain network stability [6, 18–20].

Several lines of evidence link RA to depression. Most recently, Suzuki and colleagues [21] could show that the inactivity-dependent up-regulation of synaptic efficacy mediated through RA has a rapid, antidepressant-like effect in mice similar to the antidepressant effects of ketamine. A study by Mulvey and Dougherty [22] suggests that major depressive disorder (MDD) -associated functional single-nucleotide polymorphisms (SNP) share a transcription regulation system that is activated by retinoid transcription factors and thus highly responsive to regulation through RA. Post-mortem studies could show that RA signaling, transport, and metabolism machinery is expressed in the adult hippocampus, hypothalamus, and prefrontal cortex [23–26] and that mRNA expression profiles differ between patients with mood disorders and control subjects [27, 28]. Last but not least, a causal link between dysregulation in RA homeostasis and depression is suggested by findings on the depressogenic effects of exogenous RA—as often seen in dermatological treatment with systemic retinoids [29].

Endogenous retinoid levels in neuropsychiatric disorders have thus far only been investigated by a few studies. Yang et al. [30], in their prospective study, identified reduced RA serum levels as a risk factor for the development of post-stroke depression at three months. Guo et al. [31] found reduced retinol levels in children with autism and meta-analytical evidence suggests RA dysregulation in Alzheimer’s [32]. In our previous work, we identified reduced levels of RA and retinol as well as a dysregulation of retinoid homeostasis-associated genes in patients with schizophrenia [33].

The present study is the first to investigate endogenous retinoid homeostasis parameters in a cohort of well-defined patients with MDD and matched healthy controls. Based on previous evidence, we hypothesized that retinoid homeostasis would be altered in MDD patients as compared to controls. We assessed retinoid homeostasis through several parameters. Serum levels of the biologically most active RA isomer, *all-trans* retinoic acid (*at*-RA), and its precursor ROL were analyzed and the functional signaling activity of serum RA was assessed via reporter cell assays. The individual in vitro *at-*RA metabolism activity was assessed in peripheral blood-derived mononuclear cells (PBMC). Additionally, we assessed mRNA expression profiles of several RA homeostasis-related genes such as proteins involved in retinoid transport, signal transduction, and select enzymes essentially involved in local *at-*RA synthesis and degradation.

## Methods and materials

### Participants

Patients with MDD from our in- and outpatient units and healthy control subjects matched for age, BMI, and smoking status were recruited for the clinical observational study on RA homeostasis in neuropsychiatric disorders (RAHND; ClinicalTrials.gov Identifier: NCT02439099) between 2015 and 2019. All patients received standard care treatment, had a specialist-confirmed ICD 10 diagnosis of moderate or severe depression, and did not currently receive psychotropic medication. Participants who showed any clinical signs of acute inflammation were treated with retinoids or anti-inflammatory medication or had a diagnosed neurological, somatic, or additional severe psychiatric disorder were excluded. All participants were thoroughly informed and gave their written consent. The study was approved by the local ethics committee (EA4/002/13).

### Blood samples

Samples of peripheral venous blood were obtained during a routine blood draw at admission for serum analysis of retinoid levels and isolation of PBMC. All samples were collected in a standardized manner before noon. There were no restrictions on prior meal intake. Serum aliquots were stored at −80 °C until further analysis. PBMC were isolated from heparinized blood by the FICOLL^TM^ density gradient centrifugation, according to previously established protocols [34].

### Extraction of retinoids from serum

Retinoids were extracted from sera using a liquid-liquid extraction protocol. To avoid photoisomerization, all experiments were carried out under dim red light. Serum samples were spiked with the synthetic retinoid *all-trans* acitretin as an internal standard (FC 1 µM) to assess recovery and account for inter-assay variability and to calculate final retinoid serum concentrations. Acidified ethanol and 2 vol hexane were added to 1 vol of serum. Samples were vortex mixed, shaken for 15 min, and then centrifuged at 4 °C, 1560×*g* for 5 min. The organic phase was carefully transferred into glass tubes and evaporated to dryness under a stream of argon. The same extraction procedures were applied to standard solutions for the determination of matrix effects. Dried extracts were reconstituted in HPLC eluent and centrifuged for 5 min at 21,000×*g* at 4 °C. An aliquot of 100 µl was injected into the HPLC system.

### Preparation of crude microsomal fractions for RA metabolism assays

PBMC is a widely used model to study person-specific cell dynamics and is increasingly used as a source of biomarkers for diagnostics and prediction [35–37]. The crude microsomal fractions derived from the individual PBMC contain metabolic enzymes like CYPs that are involved in the turnover of drugs or endogenous substrates such as retinoids and are thus often used to study metabolic pathways. Metabolically active crude microsomal fractions were prepared in Dulbecco’s phosphate buffered saline without Mg^++^ and Ca^++^ (pH 7.3; Gibco, Thermo Fischer) from participants’ PBMC by homogenization and differential centrifugation steps according to previously published protocols [33]. Protein concentration was determined with the BCA method (Thermo Fischer, USA) and microsomal fractions were stored at −80 °C. To determine individual RA metabolic activity, in vitro assays containing the RA-metabolizing microsomal preparations from participants’ cells were performed according to previously published protocols with minor adjustments [38]. In brief, heat-inactivated controls and metabolically active reactions contained microsomal preparations at a protein concentration of 250 µg/ml. For RA synthesis assays, ROL (FC 100 µM) and NADP+ were added to the reaction. *at-*RA (FC 2 µM) and NADPH Regeneration System (NRS; Promega, USA) were added for RA catabolism assays. After an incubation period of 60 min at 37 °C, the reaction was stopped by adding 4vol ice-cold methanol. Samples were then centrifuged at 21,000×*g* at 4 °C and subjected to HPLC retinoid analysis. *at*-RA synthesis or degradation was quantified by comparing metabolically active samples with heat-inactivated controls.

### High-performance liquid chromatography

Retinoid analysis was performed on a reverse-phase Agilent 1100 HPLC system equipped with a binary pump, temperature-controlled column compartment, auto-sampler, and a high-sensitivity diode array detector (DAD). Separation was achieved using a Phenomenex Synergi™ 4 µm Hydro-RP 80 A (250 mm × 4.6 mm) column at room temperature. The mobile phase consisted of H_2_O + 0.1% formic acid (eluent A) and acetonitrile + 0.1% formic acid (eluent B). For serum analysis, a combination of isocratic and gradient elution was used as described in Table 1 of the Supplement. At a constant flow rate of 1.7 ml/min, the total runtime was 24 min for one sample. This setup allowed for excellent resolution of retinal, ROL, RA isomers, and oxidation products (Supplementary Fig. 1). In a single HPLC run, several compounds could be clearly separated in serum, with a retention time of 5.1 min for the internal standard acitretin, 9.3 min for *at*-RA, and 10.9 min for ROL (Supplementary Fig. 2). All peaks and retention times were confirmed by authentic standards of the retinoid isomers (Sigmar Aldrich). For the quantification of retinoids in the metabolism assays, isocratic elution (15% A: 85 % B) was used with a total run time of 13 min per sample.

**Table 1:** Participant Characteristics.

		Total	MDD	HC
		*N* = 116	*N* = 58	*N* = 58
*Sociodemographics*				
Age (years)			38.8 (13.5)	36.7 (12.7)
Female *n* (%)		71 (61.2)	37 (63.8)	34 (58.6)
BMI			25.2 (5.3)	23.9 (3.1)
Current Smokers *n* (%)		43	22 (37.9)	21 (36.2)
Education *n* (%)^a^				
	Low	15 (19.2)	8 (22.2)	7 (16.7)
	Medium	21 (26.9)	14 (38.9)	7 (16.7)
	High	42 (53.8)	14 (38.9)	28 (66.7)
*Clinical characteristics*				
MADRS			23.8 (5.9)	NA
	Male^b^		17.3 (10.1)	
	Female		21.6 (9.7)	
BDI-II			28.7 (8.3)	NA
	Male		22 (12.2)	
	Female		25.5 (12.3)	
HDL cholesterol, mg/dl^a^			54.4 (18.7)	62.5 (16.4)
LDL cholesterol, mg/dl			105.2 (23.4)	98.7 (28.6)
Triglyceride, mg/dl			141.3 (85.3)	129.95 (78.5)
IL-6, ng/l			2.2 (1.4)	2.06 (1.4)
CRP, mg/l			2.7 (6.4)	1.4 (1.7)
Vitamin D3 25(OH), nmol/l^a^			62.3 (30.9)	76.8 (24.7)
Vitamin D3 1,25(OH), pmol/l			129.9 (55.95)	116.1 (34.97)

Values are expressed as mean (SD) unless otherwise indicated. Group differences were examined using chi-squared tests for categorical variables and *t*-tests for continuous variables.

^a^Significantly different from healthy controls.

^b^Significantly different from female.

*NA* not applicable, *BMI* body mass index, Education low: 10th grade, medium: secondary school, high: college/university. *MADRS* Montgomery–Åsberg Depression Rating Scale, *BDI* Beck Depression Inventory, *HDL* high-density lipoprotein, *LDL* low-density lipoprotein, *IL-6* interleukin 6, *CRP* C-reactive protein.

### Real-time PCR for relative expression of retinoid-relevant genes

mRNA expression of relevant RA-homeostasis genes in PBMC was assessed by real-time PCR. RNA was extracted using Direct-zol DNA/RNA Miniprep Kit (Zymo Research) as per the manufacturer’s instructions and the sample quality was checked afterward using Nanodrop Instrument (Thermo Fisher Scientific Inc., MA, USA). Total RNA was reverse transcribed into cDNA using Revert Aid First Strand cDNA Synthesis Kit™ (Thermo Fisher Scientific Inc., MA, USA) and stored at −20 °C until further measurement. Relative quantification (ΔCt) and melting curve analysis were both carried out using the StepOne™ Real-Time PCR System software. All primers were designed and checked for their quality using the Primer-BLAST software [39]. The sequences of primers are shown in Table 2 of the supplement. The expression fold change from the target to endogenous reference genes was calculated as 2^-ΔCt^ as suggested for presenting individual data points [40]. The values were log-transformed for statistical analysis.

### Statistical analyses, power, and sample size calculations

Group differences were examined using chi-squared tests or independent samples *t*-tests depending on the level of measurement. Effect sizes are reported as Cohen’s *d*. Linear regression was used for the analysis of the relationship between retinoid serum levels and reporter assay activity. Interaction effects were tested with a two-way ANOVA. Sample size and power calculations were performed expecting minimum group differences in serum RA levels based on previously published data comparing RA serum levels in different cohorts [41]. With an even sampling ratio, a power of 95%, and *α* = 0.01, a total of 47 participants per group were needed to detect putative group differences. Data analyses were carried out with IBM SPSS version 25. GraphPad Prism version 9 (GraphPad Software, La Jolla, CA, USA) was used for data visualization.

## Results

### Participants

In total, 116 participants were recruited for the study. Seven participants (MDD = 6, HC = 1) had to be excluded due to emerging somatic or psychiatric disorders, or previously undocumented intake of psychotropic medication. Thus, 58 MDD patients and 58 healthy controls (61.2% female) were included in the study. The two groups did not differ in age, gender, or smoking status. Further demographic data and clinical characteristics are shown in Table 1. HDL cholesterol and Vitamin D3 25(OH) were higher in healthy controls compared to MDD patients. Triglyceride levels correlated significantly positively with *at*-RA (*r* = 0.597, *p* = 0.000) and ROL (*r* = 0.532, *p* = 0.000) levels while HDL levels showed a significant negative association with *at*-RA (*r* = −0.377, *p* = 0.000) and ROL levels (*r* = −0.356, *p* = 0.001).

### Retinoid serum levels

*at-*RA and ROL serum levels were quantified by HPLC in 57 unmedicated clinically depressed patients and 52 healthy control participants. The ratios of *at*-RA to ROL were calculated. *at-*RA levels ranged from 0.73 nM to 6.3 nM with a mean of 2.8 nM (SD: ±1.17) and ROL levels ranged between 0.16 µM to 3.08 µM with an average of 1.1 µM (SD: ±0.61). Neither *at-*RA nor ROL serum levels showed a correlation with age, BMI, current smoking status, or level of education.

### Altered retinol serum levels in patients with depression

**Fig. 1:**
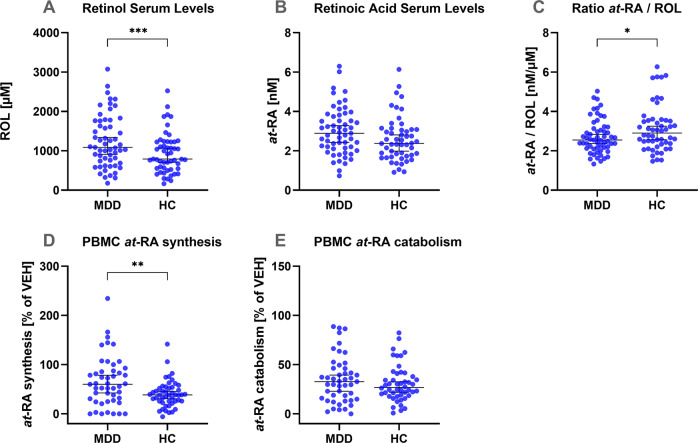
Retinoid serum levels and metabolism in patients with major depressive disorder and healthy controls. Group differences between MDD patients and healthy controls (HC) in **A** ROL serum levels. **B** *at*-RA serum levels. **C** the ratio of *at*-RA to ROL. **D** *at-*RA synthesis activity in PBMC. **E** *at-*RA catabolism activity in PBMC. All error bars depict the median with 95%CI. *t*-tests were used for group comparisons.

ROL levels were significantly higher in patients as compared to healthy controls (*t* = 2.63, *p* = 0.01, *d* = 0.5). *at-*RA levels were also higher in patients but the group difference was not significant. Moreover, the individually calculated *at-*RA/ROL ratio was significantly reduced in the patient group (*t* = −2.06, *p* = 0.042, *d* = −0.4) (Fig. 1A–C).

### Increased *at-*RA synthesis in patients with depression

To capture possible differences in retinoid metabolism, we assessed the individual in vitro *at-*RA synthesis and degradation activity in metabolically active PBMC-derived crude microsomal fractions. *at-*RA synthesis and degradation activity were assessed in standardized reactions normalized by heat-inactivated controls. The *at-*RA-synthesizing activity was significantly greater in the patient group than in the group of healthy controls (*t* = 3.022, *p* = 0.003, *d* = 0.6) (Fig. 1D). Composite scores of ROL*atRA-synthesis and RA*atRA-catabolism were built to obtain a measure of the individual homeostatic “in-“ and “out”-flow, respectively. As expected, differences in group mean of retinoid serum levels and metabolism activity was also reflected in the individual composite scores, showing a significantly increased homeostatic “in-flow” in patients as compared to healthy controls (*t* = 3.139, *p* = 0.002, *d* = 0.6).

### Expression of retinoid-relevant genes

No overall group differences were found for any of the targets.

### Gender differences in retinoid homeostasis parameters

In the patient group as well as in the control group, retinoid serum levels were significantly different in men and women. These findings together with evidence from the literature on gender differences in depression prompted us to examine group differences within gender for all retinoid homeostasis parameters. Gender differences within the group of healthy controls are also reported.

**Fig. 2:**
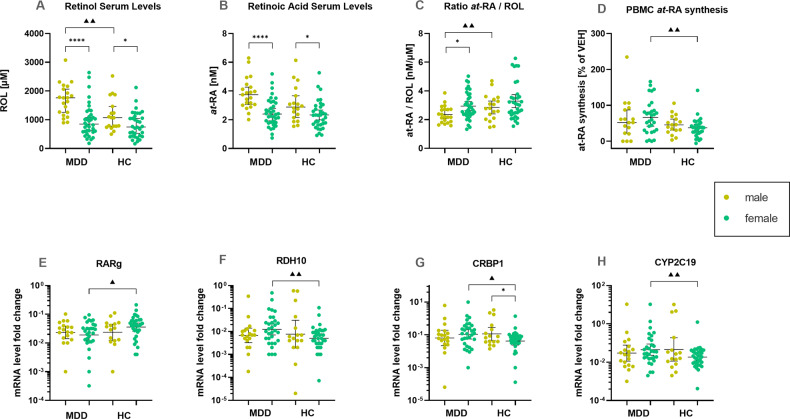
Gender differences in retinoid homeostasis parameters. * marks significant within-group gender differences (i.e., male vs female in MDD or HC). ▲ marks significant group differences for within-gender comparisons (i.e., female MDD vs female HC) **A** ROL serum levels. **B** *at*-RA serum levels. **C** the ratio of *at*-RA to ROL. **D** *at*-RA synthesis activity in PBMC. **E**–**H** Gene transcription of retinoid pathway-related genes in PBMC, fold change values presented as the 2^−ΔCt^. Error bars depict the median (**A**–**D**) or geometric mean (**E**–**H**) with 95% CI. *t*-tests were used for group comparisons.

For serum ROL levels, we found significant group differences between patients and controls for the male gender only (*t* = 2.915, *p* = 0.006*, d* = 0.9) (Fig. 2A, B). ROL levels in women did not differ between groups. Interaction effects of group × gender were not significant.

Synthesis activity in female MDD patients was significantly higher than in female controls (*t* = 3.072, *p* = 0.004, *d* = 0.8) (Fig. 2D). No such difference was found for the male gender. No significant group or gender differences were observed for RA-catabolism activity.

Differences in mRNA expression of retinoid homeostasis-relevant genes between patients and healthy controls were found only for the female gender, showing higher expression of cellular retinol-binding protein 1 (CRBP1), retinol dehydrogenase 10 (RDH10) and CYP2C19 and reduced expression of retinoic acid receptor γ (RARg) in female patients compared to female controls (Fig. 2E–H).

Within the group of healthy controls, we found gender differences in mRNA expression of CRBP1 and serum levels of *at-*RA and ROL. CRBP1 was increased in men compared to women (Fig. 2G) and *at-*RA and ROL levels were significantly higher in men than in women (Fig. 2A, B).

## Discussion

In this well-matched cohort of unmedicated patients and healthy controls, we could show for the first time that important features of peripherally assessed retinoid homeostasis, which is a key player in regulating synaptic plasticity in the central nervous system, are altered in patients with MDD. Significantly increased ROL serum levels and *at-*RA synthesis activity in depressed patients highlights a possible pathophysiological contribution of altered retinoid homeostasis.

In our own previous work on retinoid homeostasis in patients with schizophrenia, we found reduced ROL and *at-*RA levels in patients compared to controls, as well as strong effects of the atypical antipsychotic clozapine on *at-*RA levels [33]. A study by Yang et al. [30] identified reduced RA serum levels as a risk factor for the development of post-stroke depression. Both studies report a dysbalance in retinoid serum levels or metabolism activity in psychiatric patients as compared to controls, and both studies suggest a protective role of endogenous RA. Further evidence of endogenous RA in disease stems from the somatic field. Similarly, higher *at-*RA serum levels are linked to more beneficial disease outcomes for cardiovascular disease, neurological syndromes, and cancer [42–45]. A recent prospective study of 29,104 men reports reduced overall and cause-specific mortality for participants with higher ROL serum levels during a 30-year follow-up [46]. In many of the disorders investigated, increased inflammation plays a central role in the onset and disease progression. The attenuating effect of RA in these cases can be explained by its strong anti-inflammatory properties [11, 47]. Many somatic disorders are closely associated with depression by way of shared biological, social, or psychological disease pathways [48], however, retinoid homeostasis in depression has thus far not been investigated.

Interestingly, in the present study, retinoid serum levels and *at-*RA synthesis activity were increased in patients compared to controls. Especially as patients did not currently receive psychotropic medication, the statistically significant differences between patients and controls are suggested to be the result of homeostatic adaptions of the endogenous retinoid signaling system in patients with MDD. Although the meaning, direction, and extent of such adaptive processes are certainly incompletely understood, there is further evidence supporting the hypothesis of a compensatory, adaptive change within the homeostatically regulated system. *at-*RA is an important regulator and necessary for the normal functioning of a range of neurobiological processes that are implicated in depression [49]. Greater demand for *at-*RA would be reflected in increased peripheral bioavailability of its substrate ROL and increased *at-*RA synthesis activity as shown in our data (Fig. 1A, C). In the example of disturbed homeostatic synaptic plasticity, RA is produced upon chronic synaptic inactivity, to effect translational upregulation of postsynaptic glutamate receptors, leading to an upregulation of synaptic strength [6, 21]. Engaging the processes of homeostatic synaptic plasticity has also been proposed as a treatment option for MDD [3].

Throughout the analyses of the homeostasis parameters, we saw striking effects of gender. Within the group of healthy controls, *at*-RA and ROL levels were higher in men than in women, which is in line with previous findings for ROL [50]. The expression of CRBP1, which facilitates cellular uptake of serum ROL and delivers ROL intracellularly to select metabolic enzymes, was also increased in men compared to women. However, in vitro synthesis or catabolism activity did not differ between men and women. Based on our findings of gender differences in retinoid serum levels, and evidence suggesting that psychological and biological aspects of depression differ for men and women [51–53], we chose to also run within-gender comparisons of patients and controls in a secondary analysis. ROL serum levels were starkly increased in male depressed patients as compared to male controls while ROL serum levels did not differ between female patients and controls (Fig. 2A). Although other factors that we did not assess and control for might be at play, increased ROL serum levels might represent a male endophenotype of depression—or male-specific compensatory action.

The in vitro metabolism assays showed greater *at-*RA synthesis in female MDD patients than in female healthy controls (Fig. 2D). Though circumstantial and warranting further investigation, this dynamic is partially mirrored across levels of analysis in our data on mRNA expression patterns of genes relevant to RA synthesis (Fig. 2E–H). CRBP1, RDH10—one of the enzymes involved in the first of the two-step oxidation from ROL to *at-*RA—and one of the RA catabolizing enzymes, CYP2C19, were increased in female MDD patients compared to female controls, suggesting increased turnover of both *at-*RA and its substrate ROL. While CYP2C19 activity in *at*-RA breakdown is relatively low, it is interesting to note that it is also involved in the pharmacokinetics of psychotropic drugs. Fluoxetine, a major serotonin reuptake inhibitor (SSRI), potently blocks RA catabolism by inhibiting CYP2C19 activity [54, 55]. Gender differences in the pharmacokinetics and antidepressant response to SSRIs have been described [56, 57].

Taken together, we see different manifestations of altered retinoid homeostasis for men and women. While ROL serum levels were affected in male patients, retinoid metabolism was altered for female MDD patients. Gender differences and sex-specific retinoid dynamics in adult humans are scarcely discussed in the literature. Some support for gender differences in retinoid homeostasis comes from preclinical studies showing sex-specific expression patterns of retinoid receptors, heterogeneous retinol distribution across the brain areas of males and females, and strong effects of sex hormones on RA activities [58–60].

Based on previous studies on the central role of the retinoid system for depression, we sought to elucidate a putative dysregulation of retinoid homeostasis in MDD by systematically assessing various aspects of retinoid homeostasis in unmedicated patients and healthy controls. A dysbalance in homeostasis may become apparent at any one point of the transport, binding, and metabolism activities. By assessing several parameters that govern retinoid flux and determining *at-*RA availability, we obtained a broad picture of peripheral retinoid homeostasis. As psychotropic medication can influence RA metabolism [33] and the retinoid signaling system is also linked to other psychiatric, neurological, and somatic disorders, we selected patients who did not currently take any psychotropic medication and did not have major psychiatric, neurological, or somatic comorbidities. This allowed us to investigate the MDD—retinoid associations in a “raw state”. However, the MDD population without somatic or psychiatric comorbidities only represents a part of the MDD spectrum, possibly limiting the generalizability of our findings to a subgroup of MDD patients. For some group comparisons, the differences reported are rather small, yet statistically significant. Whether they are of clinical relevance remains to be investigated as there is currently no definition of minimally clinically important differences for these retinoid parameters in an MDD cohort. However, as this is an exploratory study, we chose to report even small differences between groups, as they may help to clarify the hypothesis of the involvement of retinoids in depression. Furthermore, the cohort was not originally powered to investigate gender differences. The gender distribution in this study mirrors the gender distribution of MDD in the general population, however, the sample size of male participants may have been too small to detect subtle group differences in mRNA expression or metabolism activity. Taking gender into account, future studies should assess retinoid homeostasis longitudinally to investigate the effects of treatment and compare homeostasis parameters in remitted patients to healthy controls. In addition, exploring the relationship between cholesterol and the retinoid system in depression might be an interesting avenue for future research. Given that RA is involved in lipid metabolism [61, 62], our results on significantly lower HDL levels in MDD patients, and the strong correlation of triglyceride and HDL levels with retinoid serum levels provide preliminary insights that should be evaluated further.

In conclusion, next to group differences in ROL serum levels and at-RA synthesis activity, our data suggest that MDD-associated alterations in retinoid homeostasis differ between men and women. Relations among the different homeostasis parameters as well as their relation to depression pathophysiology are highly intricate. Our study was not designed to mechanistically assess these relations. Instead, we cross-sectionally assessed several homeostasis parameters which can serve as a demonstration that investigating retinoid signaling as a putative pathophysiological mechanism of depression might be worthwhile for achieving a better understanding of the disease’s underlying neuropathophysiology.

## Supplementary information

Supplement
