# Supplementary material for: Retinoid homeostasis in major depressive disorder
Source: Transl Psychiatry. 2023 Feb 23;13:67. doi: 10.1038/s41398-023-02362-0 (PMC9947135; doi:10.1038/s41398-023-02362-0)
Supplement: Supplementary file 1 — Supplement [file 41398_2023_2362_MOESM1_ESM.docx]

**Supplement**

This supplement provides information on the additional assessment of retinoic acid signaling activity via reporter cell bioassay. The results were nicely aligned with the HPLC measurement of serum. As a matter of completeness, methods and results are described here.

Furthermore, Table 1 gives details on HPLC mobile phase, Table 2 lists the PCR sequence primers and Table 3 shows all mRNA expression profiles of the retinoid homeostasis-relevant genes. Figures 1 shows a HPLC chromatograph of retinoid authentic standards and Figure 2 shows separation of retinoids in human serum.

**Retinoic Acid Reporter Cell Bioassay**

To functionally assess the overall RA signaling activity, as a complementary measure to HPLC, we performed a cell-based reporter bioassay with participants’ sera. To that aim, SIL15 RARE reporter cells developed by Wagner and colleagues were cultured according to previous protocols (1) and seeded in a 96-well plate at a density of 5*10^4^ cells per well. Medium containing either 10% participant’s serum or fetal calf serum containing various concentrations of retinoic acid (0-100 nM) was added to the cells and left to incubate over night. Cells were subsequently lysed and added to a solution containing Fluorescein-Di-3-D-Galactopyranoside (FDG) as a substrate of β-galactosidase. Kinetic measurements for fluorescein fluorescence, which are proportional to the amount of induced enzyme in the lysates, were performed and functional RA signaling was calculated using the lysates from cells treated with known standards.

*at-*RA serum levels showed a strong relation to RA signaling activity in the reporter cell bioassay *(F* = 7.919, *p* = 0.006, *R^2^* = 0.097). The signaling activity was specific to RA, as ROL serum levels did not correlate with signaling activity. Accordingly, as group differences in *at*-RA concentration were not significant in the serum analyses, no group differences were seen for RA signaling activity. No gender differences were found for functional RA signaling.

**Table 1 Composition of HPLC mobile phase for retinoid analysis in serum**

| Time (min) | | A (%) | | B (%) | |
| --- | --- | --- | --- | --- | --- |
| 0-15.5 | 15 | | 85 | |  |
| 16 | 0 | | 100 | |  |
| 20 | 0 | | 100 | |  |
| 21 | 15 | | 85 | |  |
| Solvent A: water + 0.1 % formic acid  Solvent B: acetonitrile + 0.1 % formic acid | | | | | |

**Table 2 Primer Sequence (5’ > 3’)**

| 18S | GTG TTG AGG AAA GCA GAC ATT G | /56-FAM/TGC AGA ATC/ZEN/CAC GCC AGT ACA AGA/3IABkFQ/ | | GGT CTT CAC GGA GCT TGT T |
| --- | --- | --- | --- | --- |
| ACTB | CGA GGA CTT TGA TTG CAC ATT G | /5HEX/TT GTT ACA G/ZEN/G AAG TCC CTT GCC ATC C/3IABkFQ/ | | ACT GGG CCA TTC TCC TTA GA |
| GUSB | GAC AGT GGG CTG GTG AAT TA | /56-FAM/AC CTG TTC A/ZEN/A GTT GGA AGT GCG TCT /3IABkFQ/ | | CAT TCG CCA CGA CTT TGT TT |
| GAPDH | ACA TCG CTC AGA CAC CAT G | /5HEX/AA GGT CGG A/ZEN/G TCA ACG GAT TTG GTC /3IABkFQ/ | | TGT AGT TGA GGT CAA TGA AGG G |
| RARA | ACA CTA CGA ACA ACA GCT C | | TCC ACA GTC TTA ATG ATG CAC | |
| RARB | TGA TGG AGT TGG GTG GAC TT | | CGG ACT CGC AGT GTA GAA AT | |
| RARG | GGT TTG GGA GAA AAT GTG TCG | | GAG TCG CTC CTT ATT GGT GG | |
| RXRG | CAT GAA GAG GG AAG CTG TG | | CCA CTG GTA GCA CAT TCT G | |
| RDH10 | TAG CCG ATG TTA CTG TCC CT | | GGG AAC ATT AGC ACA CAC CA | |
| ALDH1A1 | GTG GCA AGA AAT TTC CTG TC | | TCA ACA TCC TCC TTA TCT CCT | |
| ALDH1A2 | GTT ACA ATG CCT TAA ATG CCC | | AGC CAA ATT CTC CCA TTT CTC | |
| ALDH1A3 | TAC AAC GCC CTC TAT GCA C | | CAA AGC GTA TTC ACC TAG TTC TC | |
| CRBP1 | TGG CAT CAC TTC CAG CAT TAT T | | TAA ACT GAC CCT TGA CTG AGC | |
| CYP26A1 | GAA GAG TAA GGG TTT ACT TTG C | | CCC GAT GTA TTT AAG TTG TTC C' | |
| CYP26B1 | TGG TGG AGA TGG AAT GTG GC | | TTG CTC CCC GTC AGA AGA AC | |
| CYP26C1 | ACT GGT TAG TTC AGG GCT C | | TGA ACA CTG TCC CAT AGC G | |
| LRAT | GTT CCT TAT CCG TCT CAT TCC C | | CAG GAT GTT AGC TCC GTA GG | |
| STRA6 | AAC TGC CGA GAC CAC ACA AC | | ACG ACA TTC TCT GGC CCT TC | |
| CRABP1 | GGC TTG CTC CTA CTT TCA GG | | TAG GGA TAC AAG AGG CAC CA | |
| CRABP2 | CAC CAC AGA GAT TAA CTT CAA GG | | TTC ACC AGG CTC TTA CAG G | |
| CYP1A1 | CTG AGG TCC TGA TAA GCA C | | AAC TTC TCA TTC AGG TCC TG | |
| RDH5 (RDH1) | CTG TAG GTC ACT TGG GCT CC | | TAA CGT GCA TCT CCA CCC AC | |
| RAI | TGC CGT AGT AAT CCA CCC CA | | AAC GGG TTT TCC TCC CAA CC | |
| CYP2C8 | GCA GTT ACC AAA GGG ATT GTT T | /56-FAM/TG AAG AAT G/Zen/C TAG CCC ATC TGG CT/3IABkFQ/ | | GAG TTG CAG GTG ATA GCA GAT |
| CYP2C9 | TCC CTT CCC TGA AGA TCT AGT G | /56-FAM/TC GAC CTC C/Zen/A TTA CGG AGA GTT TCC T/3IABkFQ/ | | GCA ACT GTT ACA GAG TAT GGA GAA |
| CYP2C19 | TGT CTG AAG AAG CAC AGA TGG | /56-FAM/TT TGG ACC A/Zen/G AGG AAA GAG AGC TGC /3IABkFQ/ | | GGT CAG AAG AAG CAT CAC AGA TA |

**Table 3 mRNA expression of genes relevant to retinoid homeostasis**

|  | **MDD** | | | | **HC** | | | |
| --- | --- | --- | --- | --- | --- | --- | --- | --- |
|  | **male** | | **female** | | **male** | | **female** | |
|  | Mean | N | Mean | N | Mean | N | Mean | N |
| **RARa** | 0,3712 | 19 | 0,5128 | 31 | 0,4751 | 16 | 0,4175 | 33 |
| **RARb** | 0,2512 | 19 | 0,4324 | 31 | 0,8416 | 16 | 0,0974 | 33 |
| **RARg** | 0,0323 | 18 | 0,0302 | 28 | 0,0363 | 15 | 0,0502 | 33 |
| **RXRg** | 0,0174 | 19 | 0,0265 | 31 | 0,0349 | 16 | 0,0067 | 33 |
| **RDH5** | 0,2886 | 18 | 0,2919 | 30 | 0,2365 | 16 | 0,0696 | 33 |
| **RDH10** | 0,0265 | 19 | 0,0423 | 31 | 0,0931 | 16 | 0,0107 | 33 |
| **ALDH1A1** | 0,0337 | 19 | 0,0229 | 31 | 0,0323 | 16 | 0,0158 | 32 |
| **ALDH1A2** | 0,0013 | 17 | 0,0015 | 25 | 0,0011 | 13 | 0,0011 | 33 |
| **ALDH1A3** | 0,0006 | 17 | 0,0019 | 25 | 0,0005 | 13 | 0,0003 | 27 |
| **CRBP1** | 0,4611 | 19 | 0,5504 | 31 | 0,5223 | 16 | 0,1019 | 33 |
| **CRABP1** | 0,0115 | 19 | 0,0137 | 31 | 0,0185 | 16 | 0,0047 | 32 |
| **CRABP2** | 0,0041 | 18 | 0,0044 | 29 | 0,0036 | 13 | 0,0042 | 33 |
| **LRAT** | 0,0762 | 19 | 0,1057 | 31 | 0,0428 | 16 | 0,0209 | 33 |
| **STRA6** | 0,3160 | 19 | 0,4076 | 31 | 0,6586 | 16 | 0,1213 | 33 |
| **RAI** | 0,0618 | 19 | 0,0101 | 31 | 0,0640 | 15 | 0,0127 | 33 |
| **CYP1A1** | 0,0006 | 14 | 0,0021 | 25 | 0,0041 | 16 | 0,0009 | 31 |
| **CYP2C8** | 0,6814 | 19 | 0,2797 | 31 | 1,5095 | 16 | 0,3343 | 33 |
| **CYP2C9** | 0,7689 | 19 | 0,2673 | 31 | 1,2473 | 16 | 0,0425 | 33 |
| **CYP2C19** | 0,6105 | 19 | 0,4626 | 30 | 1,2288 | 16 | 0,0600 | 33 |
| **CYP26A1** | 0,3624 | 19 | 0,4047 | 31 | 0,5633 | 16 | 0,1084 | 33 |
| **CYP26B1** | 0,5735 | 19 | 0,7735 | 31 | 0,5824 | 16 | 0,2395 | 33 |
| **CYP26C1** | 0,0040 | 19 | 0,0046 | 31 | 0,0066 | 16 | 0,0014 | 33 |

Values are expressed as fold change from the target to endogenous reference genes.

**Fig. 1 Separation of various retinoids by HPLC**

**
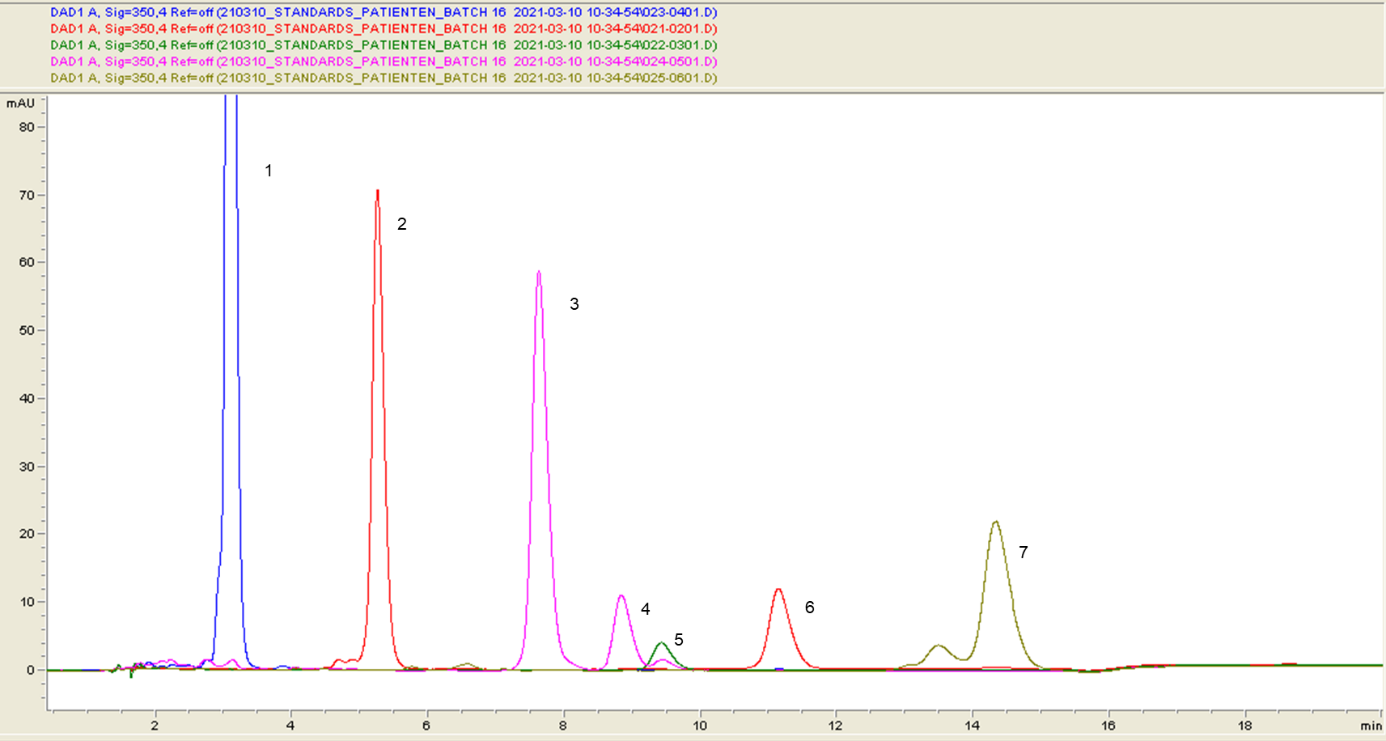
**

Retinoid authentic standards in running buffer. [1] 4-*oxo*-RA 1µM; [2] acitretin 1µM; [3] 13-*cis*-RA 1µM; [4] 9-*cis*-RA 1µM; [5] *at*-RA 100nM; [6] ROL 1µM; [7] RAL 1µM

**Fig. 2 Detection of retinoids in serum**


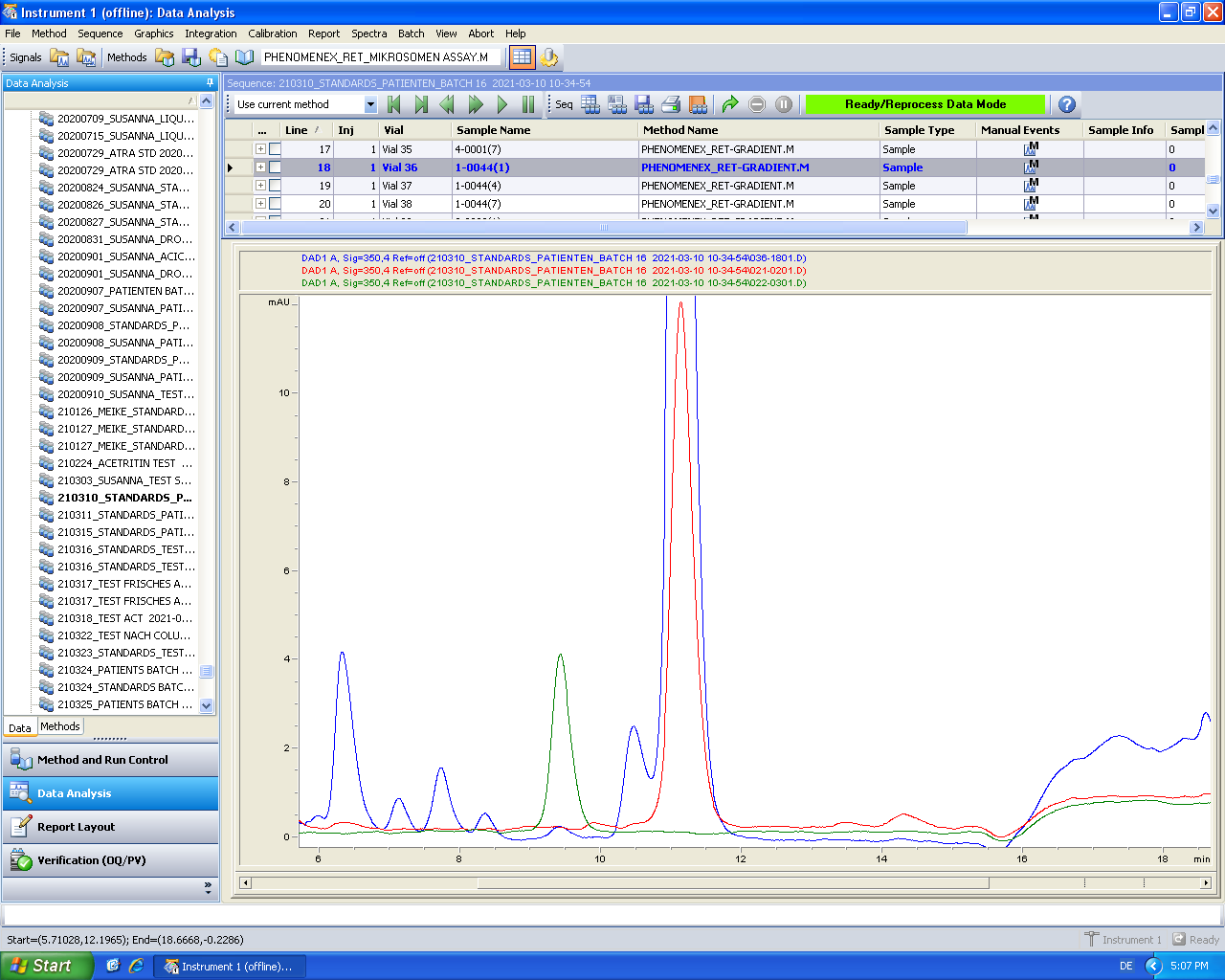


Chromatograph of a serum sample (blue line), *at*-RA 100nM authentic standard in running buffer (green line) and ROL 1µM authentic standard in running buffer (red line).

References:

1. Wagner M, Han B, Jessell TM. Regional differences in retinoid release from embryonic neural tissue detected by an in vitro reporter assay. Development. 1992;116:55–66.
